# Response Surface Analysis of Thermo-Hydraulic Performance of a Direct Absorption Solar Receiver Using Fe_3_O_4_ Nanofluid Under Concentrated Solar Irradiation

**DOI:** 10.3390/nano16100578

**Published:** 2026-05-08

**Authors:** Jeonggyun Ham, Hyemin Kim, Sang-Bum Ryu, Honghyun Cho

**Affiliations:** 1 Department of Mechanical Engineering, Chosun University, 10 Chosundae 1-gil, Dong-gu, Gwangju 61452, Republic of Korea; hhcho@chosun.ac.kr; 2 Graduate School of Chosun University, Chosundae 1-gil, Dong-gu, Gwangju 61452, Republic of Korea; kims5642@naver.com; 3 SOO ENERGY Co., Ltd., 15-31, Geogukbawi-ro 3-gil, Jinan-eup, Jinan-gun 55427, Republic of Korea

**Keywords:** direct absorption solar collector, nanofluid, response surface methodology, spiral channel, thermo-hydraulic performance

## Abstract

This study investigates the thermo-hydraulic performance of a spiral-channel direct absorption solar collector (DASC) using Response Surface Methodology (RSM). The objective of this study was to establish a design framework that balances thermal efficiency ($\eta_{th}$) and pressure drop (ΔP) by resolving inherent trade-offs. The results indicate that geometric parameters primarily influence hydraulic resistance, while optical factors govern thermal capture. To identify a robust operating region, a feasible design window was established under the constraints of $\eta_{th}\;\geq$ 0.90 and ∆P < 200 Pa. Analysis reveals that a minimum receiver height (Factor B) of 12.1 mm and a nanoparticle concentration (Factor A) of at least 0.052 wt% are required to satisfy the performance criteria. Within this identified space, an operating range of B = 18–24 mm and A = 0.06–0.08 wt% is recommended at fixed values of Factor C = 3 and Factor D = 0.59. This region-based approach provides a design foundation that respects thermodynamic limits while minimizing parasitic losses, offering practical guidelines for the optimization of spiral-channel DASC configurations.

## 1. Introduction

Improving the efficiency and operational stability of solar thermal collectors is a primary objective for expanding the presence of renewable energy [1]. Due to their inherent exposure to the environment, these systems are susceptible to heat losses that reduce overall thermal performance and reliability [2]. Specifically, configurations with large absorption areas and high ambient contact are prone to significant degradation caused by convective and radiative losses. Therefore, new receiver designs are required to minimize these losses while maximizing energy absorption.

Concentrated solar systems address these challenges by locally intensifying solar radiation, which allows for the miniaturization of receiver structures [3,4,5]. A reduced receiver area decreases the heat exchange surface with the ambient air, thereby effectively limiting convective and radiative heat losses. This configuration is advantageous for maintaining stable thermal performance despite variations in operating conditions. In particular, when water is used as the working fluid, these structural features help mitigate outlet temperature fluctuations and ensure a consistent thermal supply. Consequently, various design approaches [4,6,7,8,9] have been proposed to enhance the heat transfer performance of concentrated receivers, focusing on strategies that simultaneously optimize internal energy absorption mechanisms and flow characteristics.

The direct absorption solar collector (DASC) is based on the concept of volumetric absorption, where solar radiation is absorbed directly within the working fluid rather than on the absorber surface [10]. This mechanism disperses radiative energy throughout the flow, effectively mitigating local heat concentration and expanding the heat transfer area. When integrated with concentrated receivers, the DASC facilitates a more uniform distribution of concentrated solar energy across the fluid, which can alleviate internal temperature gradients and improve overall thermal stability [10,11]. To implement these characteristics in practical systems, both the optical absorption performance of the working fluid and the internal flow structure of the receiver must be considered simultaneously. Accordingly, the application of nanofluids has been investigated to enhance radiation absorption and heat transfer characteristics, alongside studies on internal geometry improvements to control flow mixing and energy distribution [5,12,13,14,15,16]. While nanofluids have been employed to increase volumetric absorption efficiency [14,15,16], geometric designs have evolved to effectively couple the spatial distribution of radiative energy with convective heat transfer [5,12,13].

Research on DASCs has evolved toward improving heat transfer efficiency through volumetric radiation absorption within the working fluid. Early studies focused on validating the feasibility of the direct absorption concept, while subsequent efforts have actively utilized nanofluids to enhance optical absorption coefficients and heat transfer characteristics. In particular, the influences of nanoparticle concentration, particle properties, and flow conditions on thermal collection performance have been the primary subjects of analysis [17,18,19]. Specifically, Tran et al. [20] evaluated the photothermal performance of diamond nanofluids (DNFs) synthesized via cavitation in DASCs. The experimental results showed that the volumetric absorption of DNFs significantly improves efficiency, reaching a maximum of 87.2% at a concentration of 0.25 wt%. This represents a 40.7% enhancement over distilled water, demonstrating the superior performance of DNFs as a working medium compared to conventional surface-based collectors. Haeri et al. [21] investigated TiNML125 and MXML125 hybrid nanofluids synthesized via a solvothermal process for solar energy harvesting in DASCs. Experimental results showed that TiNML125 and MXML125 achieved peak photothermal efficiencies of 86.0% and 96.8% at a concentration of 60 ppm, respectively. Both hybrid nanofluids significantly outperformed the single-component ML125, with TiNML125 improving absorption by 40% and MXML125 demonstrating superior long-term stability and heat distribution. Huminic et al. [22] investigated the outdoor performance of full-scale DASC prototypes using an Ag + rGO hybrid nanofluid dispersed in a water–ethylene glycol solution. Compared to the base fluid, the hybrid working medium enhanced the relative efficiency by up to 16.72% and increased the accumulated energy output by 42.91% at a flow rate of 1.0 L/min, highlighting its effectiveness for practical solar energy harvesting.

Concurrently, research on applying the direct absorption principle to concentrated solar thermal collectors (CSTCs) has been expanding. Vijayaraghavan et al. [23] experimentally investigated a Fresnel-lens-based concentrating direct absorption collector using a CuSO_4_ solution as a spectrally selective absorbing fluid and reported that the flow rate and optical concentration ratio had limited effects on thermal efficiency within the tested range. Kaluri et al. [24] developed a three-dimensional CFD model for a direct absorption concentrating collector by coupling the RTE with the Navier–Stokes equations and showed that increasing the optical concentration ratio improved collector efficiency by up to 28%, although the efficiency reached a threshold beyond a certain absorption coefficient for a given fluid thickness. Dugaria et al. [25] numerically investigated a concentrated direct absorption receiver using SWCNH/water nanofluid and demonstrated that volumetric absorption can improve receiver efficiency when compared with conventional surface absorption. Their results also indicated that nanoparticle concentration and channel depth strongly affect radiation penetration depth, temperature distribution, and thermal performance. Qin et al. [26] investigated the thermal impact of corrugated substrates in DASCs using finite element numerical simulations. Their results indicated that the Nusselt number in the volumetric absorption mode is 1.47–2.8 times higher than that in the surface absorption mode, with the corrugated geometry effectively mitigating re-radiation losses. Furthermore, they identified the transition from total volume to bottom absorption as the most efficient mode, resulting in a collector efficiency increase of up to 10%. In related concentrated solar receiver studies, Aslfattahi et al. [27] showed that receiver geometry and nanofluid concentration can influence both thermal efficiency and pumping work in a solar dish collector, highlighting the importance of evaluating thermal enhancement together with hydraulic penalty. Although these studies have demonstrated the potential of liquid-based and nanofluid-based volumetric absorption under concentrated irradiation, research on concentrated DASCs remains relatively limited when compared with that on conventional or low-temperature DASCs. Existing studies have primarily examined optical absorption behavior, radiative heat transfer, or thermal performance improvement, whereas the hydraulic penalty associated with receiver geometry has received less attention. In particular, the coupled thermo-hydraulic behavior of concentrated DASCs has not been sufficiently investigated when nanofluid concentration and receiver geometry are varied simultaneously.

In concentrated direct absorption receivers, the overall performance is determined by the coupled effects of the photothermal working fluid and the internal receiver geometry. Nanofluid concentration directly governs the volumetric absorption of incident solar radiation, whereas receiver height and flow-path configuration influence residence time, internal mixing, and hydraulic resistance. Therefore, enhancing solar absorption alone does not necessarily guarantee improved system performance because thermal enhancement can be accompanied by increased pressure drop or non-uniform heat transfer characteristics. Under these multivariate conditions, analyzing the influence of each design variable independently is insufficient to fully characterize the nonlinear thermo-hydraulic behavior of the receiver. A quantitative framework is therefore required to evaluate variable sensitivity, interaction effects, and feasible operating conditions. Fe_3_O_4_ nanofluid was selected as the working medium for this study because of its practical advantages for concentrated DASC applications. Fe_3_O_4_ nanoparticles exhibit strong optical absorption in the visible region, which is beneficial for volumetric solar energy absorption. In addition, their magnetic properties can facilitate the recovery or removal of deposited particles using an external magnetic field, providing an advantage in terms of system maintenance. Compared with carbon-based nanofluids, which may be susceptible to agglomeration under high-flux irradiation and elevated-temperature conditions, Fe_3_O_4_ nanofluid can offer favorable dispersion stability at high temperatures. These characteristics make Fe_3_O_4_ nanofluid a suitable photothermal working medium for concentrated direct absorption solar receivers.

Accordingly, this study employs Computational Fluid Dynamics (CFD) to investigate the coupled effects of Fe_3_O_4_ nanofluid concentration and spiral-channel geometry on the thermo-hydraulic performance of a concentrated DASC. Response Surface Methodology (RSM) is further applied to quantify the main, nonlinear, and interaction effects of the design variables, including nanoparticle concentration, receiver height, spiral guide number, and wall emissivity. The value added by this work is its integrated treatment of a water-based Fe_3_O_4_ nanofluid and a spiral-channel receiver design under concentrated irradiation. While water-based nanofluids have been widely studied in conventional or low-temperature DASCs, their use in concentrated direct absorption receivers has been less extensively investigated, particularly in terms of the trade-off between volumetric solar absorption and hydraulic loss. Therefore, this study does not simply evaluate nanofluid photothermal performance or receiver geometry independently; rather, it establishes a unified thermo-hydraulic design framework that links optical absorption, internal flow structure, and pressure drop. Finally, a feasible design window is derived based on simultaneous constraints on thermal efficiency and pressure drop, providing practical guidelines for the design of concentrated direct absorption solar receivers.

## 2. Numerical Method

### 2.1. Description of Concentrated DASC

**Figure 1:**
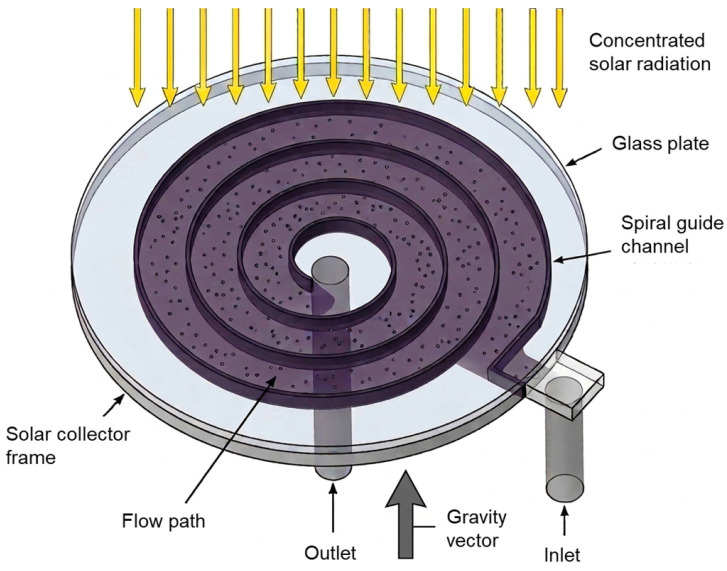
Schematic representation of a direct absorption solar collector with a spiral flow guide.

The proposed DASC was designed to address the risk of flow stagnation and localized overheating under concentrated irradiation. In a receiver without an internal flow-guiding structure, the working fluid can form stagnant or weakly circulating regions, particularly near the outer boundary or low-velocity zones. Under concentrated solar irradiation, such non-uniform flow distribution can lead to localized thermal accumulation, which may reduce thermal performance and potentially cause thermal stress or durability problems in receiver components. To mitigate this issue, a radially inward spiral flow arrangement was introduced in the present receiver. Figure 1 shows the schematic configuration and operating principle of the proposed concentrated DASC with a spiral flow guide. The receiver consists of three main components: a semi-transparent glass cover with a thickness of 3 mm, a cylindrical receiver frame with an outer diameter of 140 mm, and an internal spiral guide that defines the fluid path. Table 1 summarizes the geometric specifications and design variables of the concentrated DASC used in the simulations. Concentrated solar radiation passes through the glass cover and is absorbed volumetrically by the Fe_3_O_4_ nanofluid inside the receiver, allowing solar energy to be converted directly into thermal energy within the working fluid.

Unlike conventional longitudinal-flow receivers, the working fluid enters through an eccentric inlet located near the outer radius and follows a spiral trajectory toward the central outlet. This radially inward flow path increases the residence time of the working fluid and promotes internal energy distribution, thereby suppressing stagnant regions and reducing localized overheating. In addition, this flow arrangement helps concentrate the high-temperature fluid region near the center of the receiver, reducing the temperature level near the outer frame and limiting convective heat exchange with the ambient environment. The internal flow path is governed by the number of spiral guides ($N_{sp}$), which determines the number of revolutions, effective channel width, residence time, and hydraulic resistance. Increasing $N_{sp}$ from 3 to 7 can improve internal energy redistribution by extending the flow path, but it can also increase pressure drop owing to the reduced effective flow area and increased flow resistance. Therefore, the spiral-channel geometry must be evaluated from a thermo-hydraulic perspective that considers both thermal gain and pressure drop.

### 2.2. Numerical Model of Concentrated DASC

**Figure 2:**
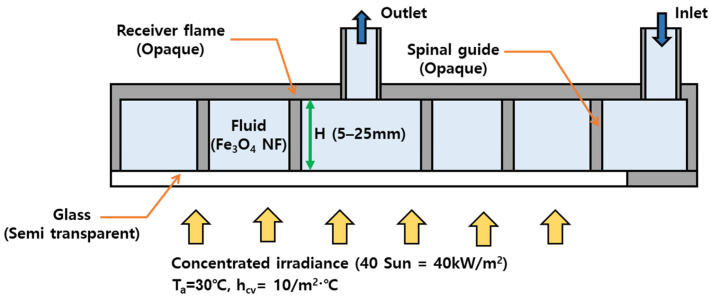
Schematic of the numerical model and boundary conditions of the concentrated DASC.

To investigate the thermo-hydraulic characteristics of the concentrated DASC using Fe_3_O_4_ nanofluid, a three-dimensional steady-state CFD simulation was performed using ANSYS Fluent 2022 R2. Figure 2 shows the numerical model and main boundary conditions adopted in the present analysis. The computational domain consists of the Fe_3_O_4_ nanofluid region, semi-transparent glass cover, receiver frame, and internal spiral guide.

Fe_3_O_4_ nanofluid was treated as an incompressible homogeneous single-phase fluid within the low-concentration range considered in this study. The Reynolds-averaged conservation equations of mass, momentum, and energy were solved to obtain the velocity, pressure, and temperature fields inside the receiver. Turbulence closure was achieved using the realizable k–ε model because the radially inward spiral flow path can induce streamline curvature, local acceleration, and recirculating flow structures within the receiver. The governing equations are expressed as follows:

(1) $$\nabla \cdot \left(\rho v\right)=0$$

(2) $$\nabla \cdot \left(\rho vv\right)=-\nabla p+\nabla \cdot \left[\left(\mu +\mu_{t}\right)\left(\nabla v+\nabla v^{T}\right)\right]$$

(3) $$\nabla \cdot \left(\rho c_{p}vT\right)=\nabla \cdot \left(k_{eff}\nabla T\right)p+S_{rad}$$

where ρ, μ, $c_{p}$ and $k_{eff}$ denote the effective density, molecular viscosity, specific heat, and effective thermal conductivity of the nanofluid, respectively. **v** denotes the velocity vector, μ_t_ denotes the turbulent viscosity obtained from the realizable k–ε model, p denotes the static pressure, T denotes the temperature, and $S_{rad}$ denotes the volumetric radiative heat source generated by the absorption of concentrated solar radiation. The radiative source term $S_{rad}$ was calculated using the non-gray DO radiation model, as described in the following section, and was coupled with the energy equation.

At the inlet, the prescribed mass flow rate and inlet temperature were imposed, while the outlet was defined as a pressure outlet. The incident solar radiation was applied to the glass surface under a concentration ratio of 40 Suns, and its spectral distribution was modeled using Planck’s blackbody relation in the radiative heat transfer model. The glass cover was modeled as a semi-transparent boundary to allow the transmission of incident radiation, whereas the receiver frame and spiral guide walls were treated as opaque surfaces with no-slip wall conditions. The pressure drop was calculated as the difference between the area-averaged static pressures at the inlet and outlet.

The pressure–velocity coupling was performed using the SIMPLE algorithm. The momentum, energy, and DO radiation equations were discretized using a second-order upwind scheme. In the coupled radiation–heat transfer calculation, the radiative transfer equation and energy equation were solved sequentially, and the DO radiation equation was updated once every 30 energy-equation iterations to reduce computational cost while maintaining numerical stability. The angular discretization of the DO model was set to 6 × 6 divisions with 3 × 3 pixels. The residual convergence criteria were specified as 10^−4^ for the continuity and momentum equations, 10^−7^ for the energy equation, and 10^−6^ for the DO radiation equation.

### 2.3. Radiative Heat Transfer Modeling

To account for the volumetric radiation absorption within the nanofluid and the coupled heat transfer (radiation, convection, and conduction), the Discrete Ordinates (DO) radiation model was employed. The DO model is particularly suitable for analyzing energy distributions in semi-transparent media, as it solves the radiative transfer equation (RTE) by discretizing it into a finite set of discrete angles. The RTE is expressed as follows:

(4) $$\nabla \cdot \left(I_{\lambda }\left(r,s\right)s\right)+\kappa_{ext,\lambda }I_{\lambda }\left(r,s\right)\;=\;\kappa_{abs,\lambda }I_{b,\lambda }+\frac{\kappa_{sca,\lambda }}{4\pi }\int_{0}^{4\pi }I_{\lambda }\left(r,s^{\prime }\right)\phi \left(s\cdot s^{\prime }\right)d\Omega^{\prime }$$

where $I_{\lambda }$ represents the radiative intensity, and $\kappa_{ext,\lambda }$, $\kappa_{abs,s}$, and $\kappa_{sca,\lambda }$ denote the spectral absorption, scattering, and extinction coefficients of the nanofluid, respectively. These are related by the fundamental identity $\kappa_{ext,\lambda }=\kappa_{abs,\lambda }+\kappa_{sca,\lambda }$.

The prescribed incident solar radiation entering the receiver was a concentration ratio of 40 Suns, and its spectral intensity distribution was defined using Planck’s blackbody relation as follows:

(5) $$I_{\lambda }\left(\lambda ,T_{sol}\right)\;=\;S_{att}\Omega_{s}\frac{2hc_{0}^{2}}{\lambda^{5}\left[exp\left(\frac{hc_{0}}{\lambda k_{B}T_{sol}}\right)-1\right]}$$

Given that solar radiation spans a wide wavelength range, a non-gray spectral band model was adopted instead of the gray-medium assumption to account for the wavelength-dependent optical properties of the nanofluid. In Equation (5), $T_{sol}$ (the solar temperature) is 5800 K, and $\Omega_{s}$ (the solid angle of the Sun as seen from the Earth) is 6.8 × 10^−5^. Also, $S_{att}$ represents the attenuation constant. The solar spectrum was divided into several wavelength bands, and the absorption coefficients of the nanofluid were independently assigned to each band to calculate the total radiative heat source. Regarding the boundary conditions, the top glass cover was modeled as a semi-transparent boundary to allow the transmission of incident solar radiation according to its optical properties, whereas the internal solid walls of the receiver were assumed to be opaque surfaces with diffuse reflection.

### 2.4. Nanofluid Modeling

The volumetric radiation absorption and thermal behavior of the DASC are governed by the integrated optical and thermophysical properties of the nanofluid. In this study, Fe_3_O_4_ nanofluid was modeled as a stable, homogeneous single-phase medium within the low-concentration range of 0–0.1 wt%. This range was selected based on the photothermal conversion mechanism of DASCs and previous observations that Fe_3_O_4_ nanofluid exhibits favorable absorption performance at approximately 0.075–0.1 wt%. Although higher nanoparticle concentrations can improve thermophysical properties and convective heat transfer, excessive particle loading can intensify scattering and reflection losses, which may reduce the effective optical absorption within the working fluid. Therefore, concentrations below 0.1 wt% were considered to ensure efficient volumetric solar absorption while avoiding excessive scattering effects.

#### 2.4.1. Optical Properties

The optical properties of the nanofluid were defined to model the volumetric absorption of incident solar radiation within the working fluid. In the low-concentration range considered in this study, the nanofluid was treated as a stable, homogeneous single-phase medium. Accordingly, the spectral extinction coefficient of the nanofluid $\left(\kappa_{ext,\lambda }\right)$ is expressed as the linear combination of the contributions from the base fluid $\left(bf\right)$ and the suspended nanoparticles $\left(np\right)$:

(6) $$\kappa_{ext,\lambda }\;=\;\kappa_{ext,bf,\lambda }\;+\;\kappa_{ext,np,\lambda }$$

For the highly transparent base fluid, scattering is neglected $\left(\kappa_{sca,bf,\lambda }\approx 0\right)$. The absorption and scattering coefficients of the nanoparticles are determined by their respective efficiencies $\left(Q_{abs,\lambda }\;\mathrm{and}\;Q_{sca,\lambda }\;\right)$ as follows:

(7) $$\kappa_{ext,np,\lambda }\;=\;\frac{3\phi }{2d_{p}}Q_{sca,\lambda },\;\kappa_{sca,np,\lambda }\;=\;\frac{3\phi }{2d_{p}}Q_{sca,\lambda },$$

where ϕ denotes the volume fraction, and $d_{p}$ denotes the nanoparticle diameter. The extinction efficiency $\left(Q_{ext,\lambda }\right)$ is the sum of the absorption efficiency $\left(Q_{abs,\lambda }\right)$ and the scattering efficiency $\left(Q_{abs,\lambda }\right)$:

(8) $$Q_{ext,\lambda }\;=\;Q_{abs,\lambda }\;+\;Q_{sca,\lambda }$$

These efficiencies are evaluated using the Rayleigh scattering approximation, which is valid for size parameters $x\;=\;\pi d_{p}n_{bf}/\lambda \ll 1$:

(9) $$Q_{abs,\lambda }=4x\cdot Im\left\{\left[\frac{m^{2}-1}{m^{2}+2}\right]\left[1+\frac{x^{2}}{15}\left(\frac{m^{2}-1}{m^{2}+2}\right)\frac{m^{4}+27m^{2}+38}{2m^{2}+3}\right]\right\}$$

(10) $$Q_{sca,\lambda }\;=\;\frac{8}{3}x^{4}{\left|\frac{m^{2}-1}{m^{2}+2}\right|}^{2}$$

where $x\;=\;\pi d_{p}n_{bf}/\lambda$ denotes the size parameter and $m\;=\;\left(n_{p}+i\kappa_{p}\right)/n_{bf}$ is the relative complex refractive index.

#### 2.4.2. Thermal Properties

The thermal behavior of the nanofluid was determined by the temperature-dependent properties of the base fluid and the intrinsic properties of the suspended Fe_3_O_4_ nanoparticles. In this study, water was used as the base fluid, and its thermophysical properties were expressed as temperature-dependent correlations to account for property variations during solar heating. In contrast, the thermophysical properties of Fe_3_O_4_ nanoparticles were treated as constant values because their variation within the operating temperature range was considered relatively small when compared with that of the base fluid. The basic thermophysical properties and correlations used in the numerical analysis are summarized in Table 2.

These properties were used as input values to evaluate the effective thermophysical properties of the nanofluid as functions of the nanoparticle volume fraction (ϕ). Although Brownian motion can contribute to thermal conductivity enhancement in nanofluids, its effect was considered limited in the present low-concentration range below 0.1 wt%. Therefore, the effective thermal conductivity was calculated using the Maxwell model [29], which represents heat conduction in a homogeneous particle suspension and accounts for the conductivity ratio between the solid particles and the base fluid:

(11) $$k_{nf}\;=\;k_{bf}\left[\frac{k_{np}+2k_{bf}+2\phi \left(k_{np}-k_{bf}\right)}{k_{np}+2k_{bf}-\phi \left(k_{np}-k_{bf}\right)}\right]$$

where $k_{np}$ and $k_{bf}$ represent the thermal conductivities of the nanoparticle and the base fluid.

The nanofluid density ($\rho_{nf}$) and the heat capacity per unit volume $\left({\left(\rho C_{p}\right)}_{nf}\right)$ are determined using the classical mixture rule [30]:

(12) $$\rho_{nf}\;=\;\left(1-\phi \right)\rho_{bf}+\phi \rho_{np}$$

(13) $${\left(\rho C_{p}\right)}_{nf}\;=\;\left(1-\phi \right){\left(\rho C_{p}\right)}_{bf}+\phi {\left(\rho C_{p}\right)}_{np}$$

where the subscripts nf, bf and np refer to the nanofluid, base fluid, and nanoparticle, respectively.

The dynamic viscosity is estimated using the Einstein model [29], which is valid for stable dispersions in the low concentration regime:

(14) $$\mu_{nf}\;=\;\mu_{bf}\left(1+2.5\phi \right)$$

### 2.5. Box–Behnken Factorial Design Modeling

To systematically analyze the relationships between design parameters and collector performance, Response Surface Methodology (RSM) was employed to analyze the thermo-hydraulic trade-off between thermal efficiency and pressure drop and to identify a feasible design window. A Box–Behnken Design (BBD) was utilized to construct the experimental matrix, as it allows for an efficient exploration of the design space with a reduced number of numerical runs. Four independent variables were selected for investigation: nanoparticle volume concentration $\left(\phi \right)$, receiver height $\left(H\right)$, number of spiral turns $\left(N_{sp}\right)$, and wall emissivity $\left(\varepsilon_{wall}\right)$. Each factor was evaluated at three distinct levels, as summarized in Table 3. A total of 25 numerical simulations were conducted to evaluate the thermo-hydraulic performance of the receiver in terms of thermal efficiency and pressure drop. The statistical significance of the main and interaction effects of the design variables was evaluated using analysis of variance (ANOVA) to verify the reliability of the developed regression models. All statistical analyses, including regression modeling, ANOVA, response surface analysis, and Pareto chart construction, were performed using PIAnO Signature.

## 3. Results and Discussion

### 3.1. Numerical Model Validation

**Figure 3:**
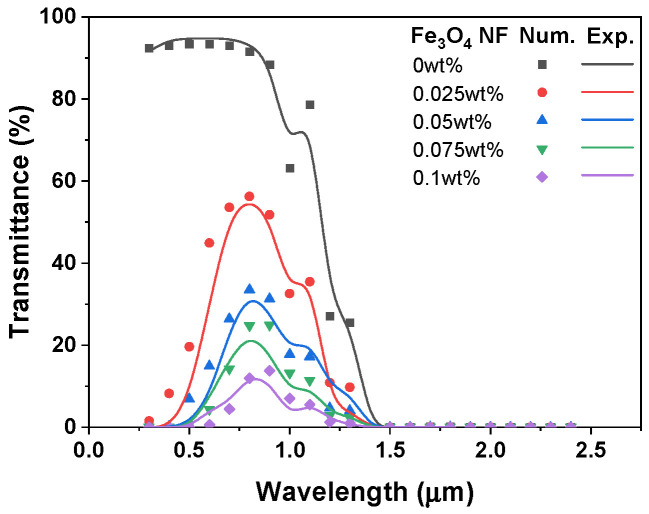
Validation of the optical model based on the spectral transmittance of Fe_3_O_4_ nanofluid.

To ensure the accuracy and reliability of the numerical results, the proposed CFD model was validated through optical property verification and a mesh independence study. First, the spectral optical properties of Fe_3_O_4_ nanofluid, calculated using the Rayleigh scattering approximation, were compared with experimental transmittance data measured using a UV–Vis spectrophotometer (AVANTES-2048, Inc. AVANTES, The Netherlands). As shown in Figure 3, the numerical model captured the overall wavelength-dependent transmittance trend for the investigated concentrations. The approximately 10% discrepancy corresponds to a local maximum deviation in a limited wavelength region where the transmittance changed rapidly, rather than the average error over the full spectral range. This result supports the use of the optical model to provide wavelength-dependent optical coefficients for the non-gray DO radiation model.

**Figure 4:**
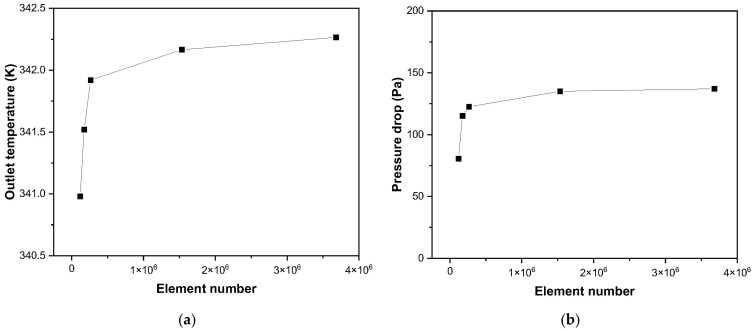
Mesh independence test based on the Run 10 geometry: (**a**) outlet temperature and (**b**) pressure drop.

Additionally, a mesh independence test was conducted to verify that the numerical results were not significantly affected by grid resolution, as shown in Figure 4. The configuration of Run 10 was used for the test, and five mesh systems ranging from 0.12 × 10^6^ to 3.68 × 10^6^ elements were examined. The outlet temperature and pressure drop were monitored as reference quantities because they correspond to the main thermo-hydraulic responses of this study. The outlet temperature is directly related to useful heat gain and thermal efficiency, while the pressure drop represents the hydraulic penalty considered in the optimization. As shown in Figure 4, both quantities showed noticeable changes in the coarse mesh region but gradually approached a converged trend with mesh refinement. When the number of elements was increased from 1.53 × 10^6^ to 3.68 × 10^6^, the outlet temperature changed by only 0.1 K, corresponding to approximately 0.03%, and the pressure drop changed by 2.01 Pa, corresponding to approximately 1.48%. These variations were considered sufficiently small for the present thermo-hydraulic analysis. Therefore, the mesh system with approximately 1.53 × 10^6^ elements was selected as the standard mesh for all subsequent simulations, considering both numerical accuracy and computational cost.

### 3.2. Evaluation of Thermal Efficiency

The statistical significance and predictive reliability of the thermal efficiency ($\eta_{th}$) model were rigorously evaluated using analysis of variance (ANOVA). To focus on the most influential parameters and enhance the model’s parsimony, a reduced quadratic model was developed by excluding non-significant terms (p > 0.1) through the backward elimination method, as summarized in Table 4. The optimized model exhibited a high F-value of 63.63 and a p-value of less than 0.0001, indicating that the regression is highly significant and the probability of the results is extremely low. The robustness of this reduced model is further evidenced by the high coefficients of determination ($R^{2}$ = 0.9591, adjusted $R^{2}$ = 0.9440), which confirm that the selected design variables effectively govern the system’s thermal behavior.

**Figure 5:**
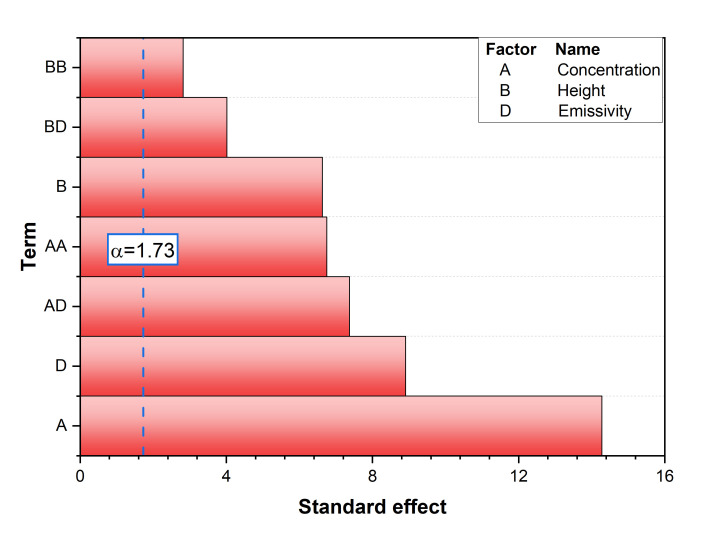
Pareto chart of the standardized effects for thermal efficiency ($\eta_{th}$).

The relative influence of each design factor on thermal efficiency is presented in Figure 5 using a Pareto chart of standardized effects. These results suggest that thermal performance in the direct absorption regime is governed primarily by volumetric radiation absorption within the nanofluid rather than by hydraulic variations induced by the spiral guide. In other words, improving the optical absorption capability of the working fluid has a stronger influence on thermal efficiency than modifying the spiral flow path within the investigated design range. The nanoparticle concentration (Factor A) was identified as the most dominant parameter, followed by wall emissivity (Factor D) and receiver height (Factor B). In contrast, the number of spiral turns ($N_{sp}$, Factor C) was found to be statistically insignificant (p = 0.628 in the full model) and was consequently removed during the model reduction process. This indicates that the volumetric radiation absorption of the nanofluid is the primary determinant of thermal performance, while the hydraulic variations induced by the spiral guides have a negligible impact on the overall energy collection in the direct absorption regime.

**Figure 6:**
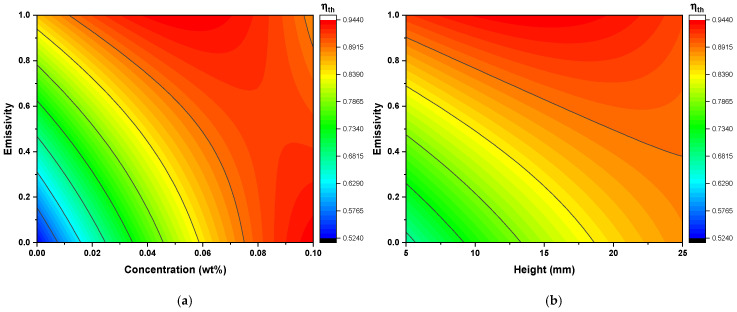
Effects of design parameter interactions on thermal efficiency ($\eta_{th}$): (**a**) ϕ−ε interaction and (**b**) H−ε interaction.

Figure 6 shows the interaction effects, specifically AD and BD, which reveal the complex thermo-optical behavior of the DASC. An increase in nanoparticle concentration (ϕ) leads to higher total energy absorption due to the increased optical thickness of the working fluid. However, the AD interaction shows that thermal efficiency is further enhanced when a high concentration is coupled with high wall emissivity. This suggests that the receiver wall facilitates the capture and re-emission of scattered radiation; a high-emissivity surface absorbs the rays scattered by the nanoparticles and reintegrates this energy into the fluid as thermal gain, whereas a low-emissivity surface would allow these scattered rays to escape through the glass cover.

The BD interaction underscores the limitations imposed by the solar penetration depth. At low concentrations, increasing the receiver height (H) extends the optical path and improves efficiency. However, at higher concentrations, the incident radiation is attenuated almost entirely near the top surface of the fluid due to concentrated scattering. In this saturation regime, the deeper fluid layers are shielded from the radiative source, rendering the increase in receiver height ineffective for further efficiency gains.

The regression equation for $\eta_{th}$ in terms of actual design parameters is derived as follows:

(15) $$\eta_{th}=0.3007+7.204A+0.01943B+0.4958D-28.51A^{2}-0.000298B^{2}-3.940AD-0.01075BD$$

The substantial negative coefficient of the $\phi^{2}$ term (−28.51) confirms the saturation effect observed at high nanoparticle loadings, where the transition from absorption to scattering-dominant regimes limits the efficiency improvement despite a larger physical volume.

### 3.3. Pressure Drop

The hydraulic performance of the spiral-channel collector was evaluated by analyzing the pressure drop (ΔP) between the inlet and outlet via numerical analysis. To accommodate the nonlinear characteristics and the wide numerical range of the pressure data, a natural log transformation was applied to the response variable, ensuring the precision and physical validity of the regression model. As summarized in the ANOVA in Table 5, the model yielded an F-value of 658.47 and a p-value of less than 0.0001, confirming high statistical significance. The coefficients of determination ($R^{2}$ = 0.9937 and adjusted $R^{2}$ = 0.9922) further indicate that the model effectively accounts for the variations in the numerical results.

**Figure 7:**
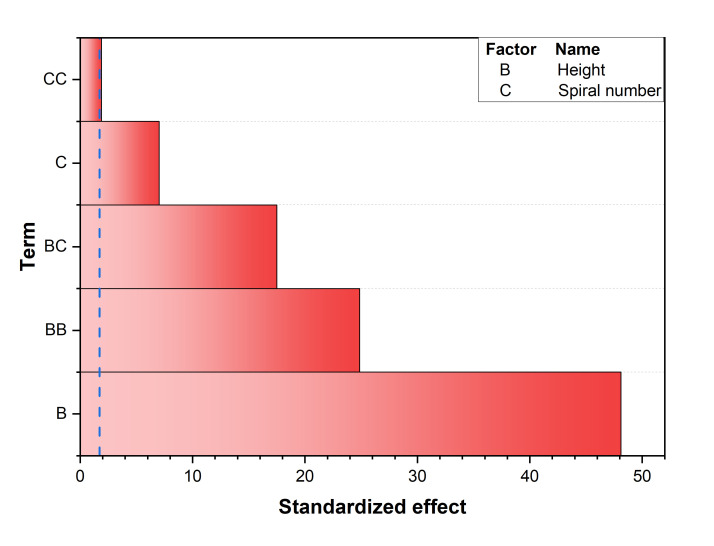
Pareto chart of the standardized effects for pressure drop (ΔP).

Figure 7 shows the Pareto chart analysis on the pressure drop. The receiver height (H, Factor B) and the number of spiral turns ($N_{sp}$, Factor C) were identified as the primary determinants of hydraulic resistance. Nanoparticle concentration (ϕ, Factor A) was excluded from the model because, within the investigated range (up to 0.1 wt%), the addition of particles did not sufficiently alter the effective viscosity to produce a measurable change in pressure drop. Similarly, the wall emissivity ($\varepsilon_{wall}$, Factor D) was found to have a negligible impact on bulk momentum transport within the numerical framework.

**Figure 8:**
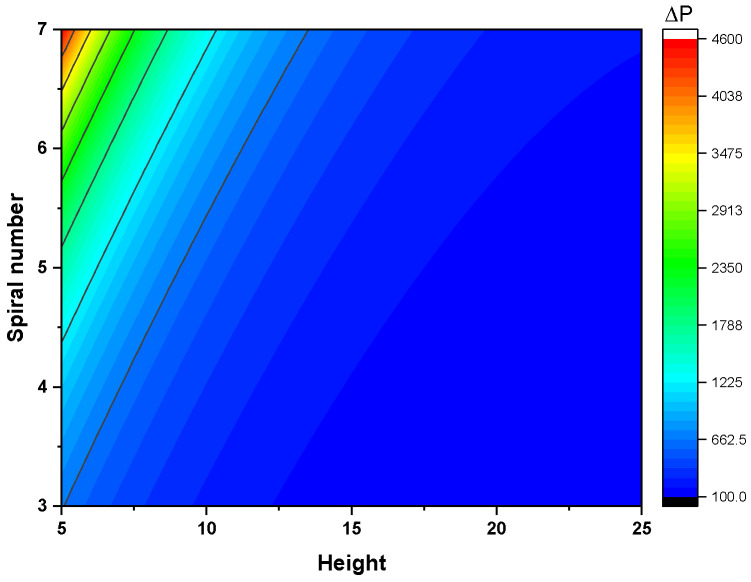
Effects of design parameter interactions on pressure drop ($H-N_{sp})$.

Figure 8 shows the effects of the BC interaction on pressure drop. The hydraulic behavior is governed by variations in flow velocity induced by changes in the channel geometry. An increase in the number of spiral turns ($N_{sp}$) constrains the individual flow width within the fixed receiver diameter, thereby reducing the cross-sectional area. This reduction intensifies the fluid velocity for a constant mass flow rate, leading to higher wall shear stress and an increased pressure drop. Conversely, increasing the receiver height (H) expands the cross-sectional area, which mitigates the pressure drop by reducing the flow velocity. The BC interaction further illustrates that the pressure-reducing effect of increasing H is more pronounced at higher $N_{sp}$ values. The final regression equation for the pressure drop is as follows:

(16) $$ln\left(\Delta P\right)=6.431-0.2317B+0.3903C+0.006200B^{2}+0.01678C^{2}-0.01576BC$$

Considering the above equation, the hydraulic performance of the DASC is predominantly governed by the geometric design of the flow path rather than the thermophysical properties of the working fluid or the optical characteristics of the boundaries.

### 3.4. Optimal Design Guidelines Based on Thermo-Hydraulic Trade-Off Analysis

**Figure 9:**
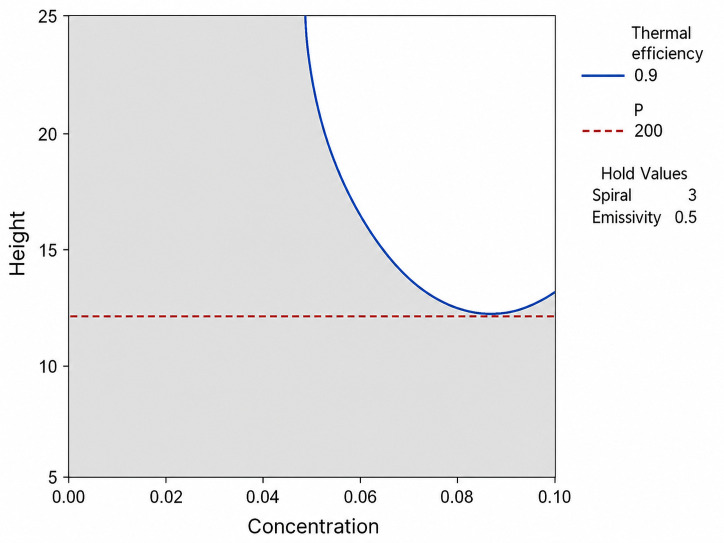
Overlaid contour plot showing the optimal design space for achieving high thermal efficiency and low hydraulic resistance.

Figure 9 presents the overlaid contour plot of thermal efficiency and pressure drop as a function of nanoparticle concentration and receiver height. The unshaded white region in the plot represents the feasible design space, where both predefined performance criteria—$\eta_{th}\;\geq$ 0.90 and ΔP ≤ 200 Pa—are simultaneously satisfied. The boundaries of this region are delineated by the solid blue line (representing the 90% efficiency threshold) and the dashed red line (representing the 200 Pa pressure drop limit). As shown in Figure 9, any design configuration falling within this white window ensures a high-density energy harvest while maintaining parasitic pumping losses at an acceptable level. Specifically, the plot illustrates that, for concentrations below 0.052 wt%, the target efficiency cannot be met regardless of the receiver height. Similarly, for heights below 12.1 mm, the system fails to satisfy the pressure drop constraint due to the intensified flow resistance. Furthermore, Figure 9 highlights the operational flexibility of the spiral-channel DASC; at a moderate height of 20 mm, a wide range of concentrations (0.06 to 0.10 wt%) can be employed without violating the design constraints. This visualization effectively captures the complex thermo-hydraulic trade-off and serves as a robust tool for determining the optimal geometric and fluidic parameters for high-performance solar collectors.

## 4. Conclusions

This study investigated the thermo-hydraulic performance of a spiral-channel DASC using Response Surface Methodology. The results indicate that pressure drop is primarily sensitive to geometric parameters, whereas thermal efficiency is governed by optical factors. To address the trade-off between these characteristics, a design window was defined under the constraints of $\eta_{th}\geq$ 0.90 and ∆P≤ 200 Pa. The analysis demonstrates that a minimum receiver height of 12.1 mm and a nanoparticle concentration of at least 0.052 wt% are required to satisfy the performance criteria. For practical implementation, an operating range of B = 18–24 mm and A = 0.06–0.08 wt% is recommended at fixed values of C = 3 and D = 0.5. This region-based approach provides a design foundation that respects thermodynamic limits while minimizing parasitic losses in spiral-channel DASC configurations.

## Figures and Tables

**Table 1:** Geometric specifications and design variables of the DASC.

Parameter	Value
Receiver diameter	140 mm
Receiver height	5/15/25 mm
Glass height	3 mm
Spiral guide number	3/5/7
Inlet/outlet diameter	10.2 mm

**Table 2:** Thermophysical properties of water and Fe_3_O_4_ nanoparticles used for effective nanofluid property modeling.

Properties	Water	Fe_3_O_4_ [28]
Density $(\mathrm{kg}/m^{3}$)	$-0.0035T^{2}+1.8142T+765.33$	5810
Specific heat (J/kg·℃)	$1.857\times 10^{-6}T^{4}-0.00248T^{3}+1.25T^{2}-281.7T+28070$	670
Thermal conductivity (W/m·℃)	$-8.151\times 10^{-6}T^{2}+0.006397T-0.5752$	80.1
Viscosity (Pa·s)	$-2.244\times 10^{-9}T^{3}+2.344\times 10^{-6}T^{2}-0.0008207T+0.0967$	

**Table 3:** Box–Behnken design matrix with coded factor levels and numerical results for thermal efficiency and pressure drop.

Run	Concentration (Level)	Height (Level)	Spiral Number (Level)	Emissivity (Level)	$\eta_{th}$	∆P (Pa)
1	0.05 (0)	15 (0)	7 (1)	0.9 (1)	0.9141	523
2	0.05 (0)	25 (1)	7 (1)	0.5 (0)	0.9055	227
3	0.1 (1)	15 (0)	3 (−1)	0.5 (0)	0.9128	135
4	0.1 (1)	25 (1)	5 (0)	0.5 (0)	0.9172	124
5	0 (−1)	15 (0)	5 (0)	0.1 (−1)	0.47	263
6	0.05 (0)	5 (−1)	7 (1)	0.5 (0)	0.787	3888
7	0 (−1)	25 (1)	5 (0)	0.5 (0)	0.7191	125
8	0.1 (1)	15 (0)	7 (1)	0.5 (0)	0.914	525
9	0.1 (1)	15 (0)	5 (0)	0.1 (−1)	0.9064	261
10	0.05 (0)	25 (1)	3 (−1)	0.5 (0)	0.8783	135
11	0 (−1)	15 (0)	3 (−1)	0.5 (0)	0.6843	136
12	0.05 (0)	5 (−1)	5 (0)	0.9 (1)	0.8989	1766
13	0.05 (0)	5 (−1)	3 (−1)	0.5 (0)	0.7793	655
14	0.1 (1)	15 (0)	5 (0)	0.9 (1)	0.9208	260
15	0 (−1)	5 (−1)	5 (0)	0.5 (0)	0.6435	1701
16	0.05 (0)	15 (0)	5 (0)	0.5 (0)	0.8792	261
17	0.05 (0)	25 (1)	5 (0)	0.1 (−1)	0.8926	124
18	0 (−1)	15 (0)	7 (1)	0.5 (0)	0.6995	525
19	0 (−1)	15 (0)	5 (0)	0.9 (1)	0.8784	260
20	0.05 (0)	15 (0)	7 (1)	0.1 (−1)	0.8452	525
21	0.05 (0)	5 (−1)	5 (0)	0.1 (−1)	0.6607	1772
22	0.05 (0)	25 (1)	5 (0)	0.9 (1)	0.9158	124
23	0.05 (0)	15 (0)	5 (0)	0.5 (0)	0.8792	261
24	0.1 (1)	5 (−1)	5 (0)	0.5 (0)	0.8454	1663
25	0.05 (0)	15 (0)	5 (0)	0.5 (0)	0.8792	261

**Table 4:** Statistical analysis (ANOVA) for the regression model of thermal efficiency ($\eta_{th}$).

Source	SS	Df	MS	F-Value	*p*-Value	Remark
Model	0.318279	7	0.045468	63.63	0	Significant
A (Concentration)	0.145649	1	0.145649	203.84	0	Significant
B (Height)	0.031382	1	0.031382	43.92	0	Significant
D (Emissivity)	0.05677	1	0.05677	79.45	0	Significant
A^2^	0.032511	1	0.032511	45.5	0	Significant
B^2^	0.005686	1	0.005686	7.96	0.011	Significant
AD	0.038806	1	0.038806	54.31	0	Significant
BD	0.01155	1	0.01155	16.17	0.001	Significant
Residual	0.013576	19	0.000715			
Total	0.331855	26				
R^2^= 0.9591, R^2^ (predicted) = 0.879, R^2^ (adjusted) = 0.844						

**Table 5:** Statistical analysis (ANOVA) for the regression model of pressure drop ($ln\left(\Delta P\right)$).

Source	SS	Df	MS	F-Value	*p*-Value	Remark
Model	26.4465	5	5.2893	658.47	0	Significant
B (Height)	18.597	1	18.597	2315.18	0	Significant
C (Spiral number)	4.9702	1	4.9702	618.75	0	Significant
BC	0.3973	1	0.3973	49.46	0	Significant
B^2^	2.4604	1	2.4604	306.3	0	Significant
C^2^	0.0288	1	0.0288	3.59	0.072	Significant
Residual	0.1687	21	0.008			
Total	26.6152	26				
R^2^= 0.9937, R^2^ (predicted) = 0.9752, R^2^ (adjusted) = 0.9922						

## Data Availability

The original contributions presented in this study are included in the article. Further inquiries can be directed to the corresponding author.
